# Impact of Circadian Rhythms on the Development and Clinical Management of Genitourinary Cancers

**DOI:** 10.3389/fonc.2022.759153

**Published:** 2022-03-09

**Authors:** Priya Kaur, Nihal E. Mohamed, Maddison Archer, Mariana G. Figueiro, Natasha Kyprianou

**Affiliations:** ^1^ Department of Urology, Icahn School of Medicine at Mount Sinai, New York, NY, United States; ^2^ Light and Health Research Center, Department of Population Health Science and Policy, Icahn School of Medicine at Mount Sinai, New York, NY, United States; ^3^ Tisch Cancer Institute, Mount Sinai Health, New York, NY, United States; ^4^ Department of Oncological Sciences, Icahn School of Medicine at Mount Sinai, New York, NY, United States

**Keywords:** prostate cancer, kidney cancer, bladder cancer, genitourinary cancers, melatonin, chronotherapy, circadian rhythm, CLOCK proteins

## Abstract

The circadian system is an innate clock mechanism that governs biological processes on a near 24-hour cycle. Circadian rhythm disruption (i.e., misalignment of circadian rhythms), which results from the lack of synchrony between the master circadian clock located in the suprachiasmatic nuclei (SCN) and the environment (i.e., exposure to day light) or the master clock and the peripheral clocks, has been associated with increased risk of and unfavorable cancer outcomes. Growing evidence supports the link between circadian disruption and increased prevalence and mortality of genitourinary cancers (GU) including prostate, bladder, and renal cancer. The circadian system also plays an essential role on the timely implementation of chronopharmacological treatments, such as melatonin and chronotherapy, to reduce tumor progression, improve therapeutic response and reduce negative therapy side effects. The potential benefits of the manipulating circadian rhythms in the clinical setting of GU cancer detection and treatment remain to be exploited. In this review, we discuss the current evidence on the influence of circadian rhythms on (disease) cancer development and hope to elucidate the unmet clinical need of defining the extensive involvement of the circadian system in predicting risk for GU cancer development and alleviating the burden of implementing anti-cancer therapies.

## Introduction

In 2017, three investigators were jointly awarded the Nobel Prize in Physiology or Medicine for their work on molecular mechanisms controlling the circadian system. The circadian system is an innate clock mechanism that governs biological processes on a near 24-hour cycle (1, 2). The evolutionary-conserved process regulates the sleep-wake cycle as well as molecular and cellular operations. The master clock is located in the suprachiasmatic nuclei (SCN) of the hypothalamus (3). The clock responds to environmental cues, such as light-dark patterns, to allow an individual to maintain synchrony with the external environment (4). In other words, through light-dark signals from the environment, the SCN is synchronized to the local position on Earth (3). In addition, clock genes in the SCN use neural signals to synchronize peripheral clocks located in the body to the external solar day (3). The circadian clock intrinsically drives transcriptional and translational feedback loops (TTFL) that regulate bodily activities (2, 5). The near 24-h cycles of gene expression are promoted by two activator clock proteins, Brain and Muscle ARNT-Like 1 (BMAL1) and Circadian Locomotor Output Cycles Kaput (CLOCK), and two repressor proteins, Period (PER) and Cryptochrome (CRY) (5). Disruption and mutation of the four integral clock proteins can misalign circadian rhythms (CRs, endogenous rhythms that are generated and regulated by then master circadian clock and repeat themselves roughly every 24 hour) such as core body temperature, hormone secretion, and sleep-wake activity (6).

Circadian rhythms disruption (CRDs; which result in misalignment of circadian rhythms, such as hormone production and the sleep-wake cycle have been shown to correlate with increased prevalence and mortality of GU cancers (7). Non-pharmacological interventions including chronotherapy and melatonin have been implicated in the treatment of CRDs. The four integral clock proteins, PER, CRY, BMAL1, and CLOCK, all have complex molecular roles that can improve our understanding of cancer risk and biologically/clinically relevant outcomes (1, 6). Yet, non-pharmacological treatments of chronotherapy and melatonin (e.g., light therapy, behavioral interventions) have diminished the toxicity of chemotherapeutic and immunotherapeutic drugs, while increasing their overall efficacy against aggressive disease (7). In this review we discuss the current evidence recognizing the significant role CRs play in GU cancer risk, development, and treatment outcomes.

## Effect of Environmental Cues on CRs

The daily light-dark pattern reaching the retina is the primary input to synchronize the biological clock to the 24-h solar day (6). If humans are not exposed to a sufficient amount of light from the right spectrum for an adequate amount of time, and with the right timing, the biological clock becomes desynchronized with the solar day, resulting in CRDs (8). CRDs are primarily caused by alterations in the circadian clock (i.e., the timekeeping system) or by a misalignment between the endogenous CR (e.g., sleep-wake cycle and hormone production) and the external factors that affect the timing, quality, or duration of sleep (e.g., sleep hygiene, environment, behavior, and social factors) (6, 8). CRDs can profoundly impact physical and daily functioning and have been linked to increased risk of insomnia, heart attacks, immune system imbalance, inflammation, diabetes, and obesity in healthy and chronic disease populations (9–11).

Recent studies confirmed associations between CRDs, increased cancer risk, and worse cancer outcomes (3, 12). Additionally, several environmental and behavioral conditions that may increase CRDs could also be independently associated with increased cancer risks (e.g., jet lag, shift work, and exposure to light at night) (12). Interestingly, a few studies showed that blind individuals with no light perception are less at risk of developing cancer (13, 14). Understanding the molecular mechanisms of the master clock in relation to its role in cell proliferation, DNA damage response, and apoptosis may provide insight into combating cancer incidence and prevalence (15).

## CRDs and Increased Risk of Genitourinary Cancer

Evidence suggests that CRDs have a role in an increased risk of cancer progression, leading to unresponsive disease, especially in endocrine-based cancers (16). In the majority of patients treated for genitourinary cancer (GU), including prostate, kidney, and bladder cancer, there is an emergence of tumor recurrence due to therapeutic resistance (17). Prostate cancer (PCa) patients are especially at risk of developing castration-resistant prostate cancer (CRPC) after initially promising therapy with androgen deprivation (ADT) (18). The androgen receptor (AR) remains a prominent driver of therapeutic resistance in PCa (19). AR variants, amplification, and mutations all serve as mechanisms of CRPC progression (19). Despite the implementation of ADT, cells can develop sensitivity to low levels of androgens and lead to treatment-resistance and recurrent fatal disease (19).

In patients with renal cell carcinoma (RCC), there is a progression to chemotherapy-resistant disease that fails to respond to tyrosine kinase inhibitors, although there is burgeoning hope with new small molecule inhibitors (20). The mechanisms of resistance to therapy in RCC are still not fully defined. However, it is hypothesized that angiogenic escape is a possible mechanism that can occur from chronic vascular endothelial growth factor (VEGF) suppression (21). Angiogenic escape involves restoring blood follow in the tumor-associated vasculature, increasing the chances of therapeutic resistance (21).

Metastatic urothelial cancer of the bladder has also been shown to be resistant to immunotherapy and chemotherapy (22). Cisplatin is a key component of chemotherapies treating bladder cancer and is the target of therapeutic resistance (23). There are many ways resistance can arise in bladder cancer, including reduced intracellular accumulation of cisplatin and increased sequestration (23). These factors all enable the cancer cells to elude the therapeutic potential of cisplatin.

## Chrono-Pharmacological Treatments of CRDs

Chronotherapy and melatonin are the two most promising non-pharmacological options to improve current anti-cancer drugs. Chronotherapy refers to the optimal dosing time of drugs where high efficacy and low toxicity are achieved (24). Time-dependent dosing relies on the oscillations of genes involved in drug absorption, distribution, metabolism, and excretion (24). Melatonin is a pineal gland hormone and is concurrently released during the hours of sleep (25, 26). However, it also possesses anti-tumorigenic abilities through an unknown mechanism of action (25, 26). Nocturnal melatonin secretion can persists in constant darkness, but exposure to light during the nighttime can suppress the release of the hormone into the bloodstream (25). The endogenous activity of the central clock results in melatonin production, so suppression of melatonin can lead to stimulation of cancer development (27). The possibility of chronotherapy and melatonin supplementation can be applied as a new platform to enhance the efficacy of chemotherapy drugs through precise time-dependent administration (28). A review by Bermu´ dez-Guzma´ and colleagues showed that melatonin, used as adjunct treatment concurrent with chemotherapy or radiotherapy, significantly improved tumor remission and 1-year survival (28). Co-administering melatonin and cancer treatments could also result in the patient having fewer adverse effects and improved outcomes (29).

## Critical Effectors of the Circadian Clock

**Figure 1:**
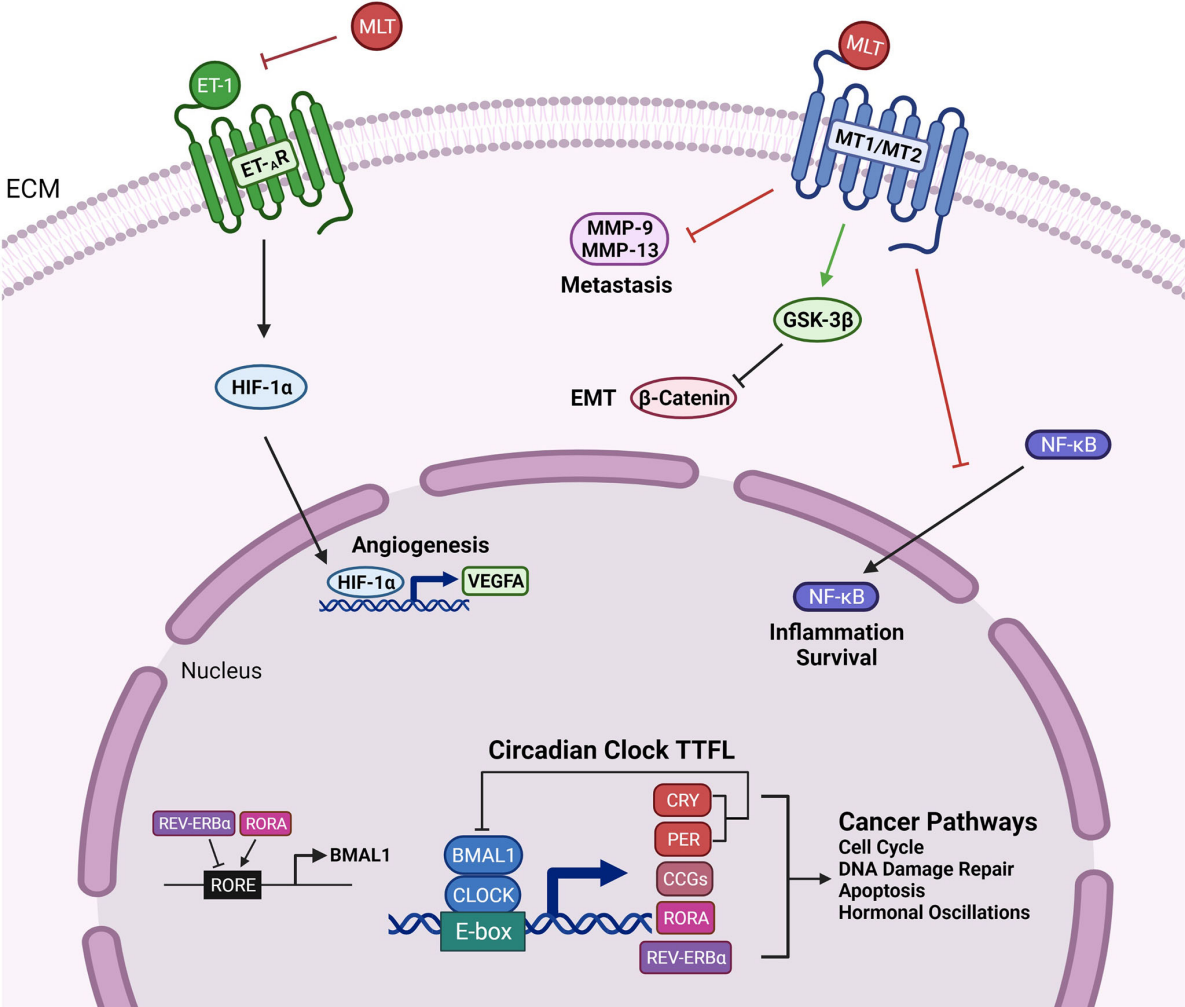
Genetic Outcomes of the Circadian Clock Proteins and Clinical Management Techniques. Circadian clock transcription-translation feedback loop (TTFL) is controlled by two activator proteins Brain and Muscle ARNT-Like 1 (BMAL1) and Circadian Locomotor Output Cycles Kaput (CLOCK), and two repressor proteins, Period (PER) and Cryptochrome (CRY). BMAL1 and CLOCK heterodimerize and bind to the E-box motif to activate the transcription of CRY (1-2), PER (1-3), clock-controlled genes (CCGs), RORα, REV-ERBα. CRY and PER establish the primary negative feedback loop by inhibiting the BMAL1 and CLOCK heterodimer. In the secondary feedback loop, RORα activates, and REV-ERBα inhibits the transcription of BMAL1. Circadian clock proteins mediate several cancer pathways such as cell cycle regulation, DNA damage repair, apoptosis, and hormonal changes. Melatonin binds to the MT1 and MT2 receptors and targets inflammation and survival pathways by preventing the translocation of NF-κB to the nucleus. Melatonin interferes with EMT and metastasis by downregulating β-catenin through activation of GSK-3β and inhibiting the expression of matrix metalloproteinases-9 and -13. The inhibition of endothelin-1 (ET-1) by melatonin leads to reduced activity of angiogenic factors HIF-1α and VEGFA.

The regulation of the CRs occurs at the transcriptional level. There are four key circadian clock proteins: BMAL1, PER (1–3), CLOCK, and CRY (1-2) (**Figure 1**) (30). Brain and Muscle Arnt-like protein, also known as BMAL1, is an integral transcription factor (31). It is a known activator of the master clock and is present in the transcriptional feedback loop (32). REV-ERBα (NR1D1) and RORα are two major nuclear receptors involved in the regulatory loop for BMAL1 (**Figure 1**) (33, 34). The heterodimer of BMAL1 and CLOCK binds to the E-box motif and activates the transcription of REV-ERBα, RORα, two repressor proteins, PER and CRY, as well as other clock-controlled genes (CCGs) (**Figure 1**) (32). CRY is known to be the primary driver of the circadian oscillator through repressing the CLOCK : BMAL1 heterodimer (**Figure 1**) (35). PER2 is the sole protein that interacts with CLOCK, whereas both PER and CRY proteins interact with BMAL1 (36). Future research on the binding and repression of the CLOCK : BMAL1 transcriptional activity will clarify the other regulatory roles of the proteins in the CRs (36).

Disruption of gene expression may lead to diseases since the clock proteins are involved in several transcriptional pathways. For instance, it was found that if the *PER2* gene is downregulated, there is an increased risk for breast cancer (37). In contrast, if the *PER2* gene is overexpressed, it may confer tumor-suppressive properties (38). In colorectal cancer, increased levels of BMAL1 have been related to decreased survival, and similarly, reduced levels of PER2 and PER3 have led to more inadequate tumor differentiation (39). Other studies have found that the clock gene expressions were reduced to 60% in melanoma and naevus tumors, highlighting their role in transcription regulation and tumorigenesis (40). With increasing evidence, research suggests that the clock proteins are also involved in genotoxic stress and aging, which are two factors that can also lead to carcinogenesis (41). Thus, disturbances of the circadian clock gene expression leading to interesting downstream effects can play a role in the carcinogenesis of various cancers.

Other factors, such as exposure to light at the wrong circadian time (e.g., exposure to ambient electric light during night shifts) or not enough light exposure at the right circadian time (e.g., not enough exposure to daylight), can alter the timing of the biological clock in humans (42). In particular, melatonin, a pineal gland hormone, can be affected by the amount and distribution of light signals picked up by the retina (43). With increased exposure to light at night, blood melatonin levels may be suppressed, leading to CRDs (43). Melatonin influences CRY1 expression, and melatonin suppression resulting from increased exposure to light at night, can compromise CRY1’s function in regulating CRs (44). Thus, electric light at night in the environment can disrupt pineal function and thus be linked to a higher incidence of hormone-related cancers such as PCa and breast cancer (43). The indirect light-induced stimulation of tumor development may be associated with the inhibitory clock proteins PER1 and PER2 (44). Specifically, disrupting PER2, CRY2, or BMAL1 in various tissues can increase the likelihood of cancer development (44). A light-induced signaling pathway is also involved in regulating the cell division cycle (44, 45). AP-1 is a transcription factor involved in maintaining biological processes, such as cell proliferation and apoptosis (45), and was found to have light-dependent activation in the SCN, adding to evidence that light plays a vital role in cancer development and circadian rhythm regulation (45).

## CRDs and GU Cancers

### Prostate Cancer

Prostate cancer (PCa) is the second most frequent cancer diagnosis made in men with 1,276,106 new cases of reported worldwide in 2018 (46). In the United States, an estimated 248,530 new cases and 34,130 deaths are estimated in 2021 (47). Although differences in PCa incidence rates worldwide reflect differences in the use of diagnostic testing and PCa screening guidelines, both incidence and mortality rates are strongly related to age with the highest incidence being seen in elderly men (> 65 years of age) (46). In the United States, PCa screening is highly recommended at age 40 for men with familial history and men of African ancestry (48).

For early stage PCa patients survival is 99% for the first five years after localized treatment (49). However, eventually, many PCa patients develop therapeutic resistance to ADT, otherwise known as castration-resistant prostate cancer (CRPC) (50). This leads to an incurable disease in which 19.5% of patients died from metastatic-CRPC in 2020 (51). There has been a recent shift to using taxane-based chemotherapy to treat CRPC patients (52). Taxanes are an excellent option for resistant PCa as they stimulate apoptosis by disrupting the G2/M-phase of the cell cycle (53). Despite the benefits of taxanes, 1^st^ and 2^nd^ line taxane chemotherapy (Docetaxel and Cabazitaxel, respectively) in patients with advanced metastatic disease, ultimately, emergence of therapeutically resistant tumors leads to lethality.

Significantly enough, disruption of CRDs have been implicated in PCa risk and progression (54). Compelling evidence suggests a significant correlation between light exposure at night and increased PCa incidence (54). Additional studies from independent investigators have exploited melatonin suppression and shift work and their positive correlations with PCa risk (55, 56). Increased risk of PCa among night male shift workers is attributed to changes in amplitude of melatonin and associated changes in sex hormone secretion that contribute to Epithelial-to-mesenchymal transition (EMT) typically involved in PCa development (55, 56). Two pathways may result in reduced amplitude of melatonin among male night shift workers; a) the acute melatonin suppression through exposure to electric light after dusk (57); and b) the decreased melatonin levels through CRDs (58), that consequentially results in desynchronization of the peripheral clocks, promoting cell growth and tumor development (58). Melatonin may suppress PCa growth by down regulating transcription, secretion, or activity of growth factors; it may stimulate the immune system through increased production of interleukin-2 and interleukin-4 by T-helper cells; lastly, it may protect DNA against oxidative damage by scavenging free radicals (58). It is thus apparent that disruption of the CRs can lead to increased PCa risk (**Table 1**). Moreover, growing evidence supports an intricate relationship between PCa, and the effector proteins functionally associated with the circadian clock. These proteins regulate cancer mechanisms such as apoptosis or proliferative cancers (58, 59). A study found that PER2 and CLOCK protein levels were downregulated, and in contrast, BMAL1 was upregulated in PCa tissue (60). Another circadian repressor protein, CRY1, is a known regulator of cell proliferation and DNA repair (61). CRY1 was upregulated in PCa and thus indicated a poor outcome for metastatic-CRPC (61). Like many clock proteins, CRY1 has transcriptional control aside from its role in regulating the circadian clock (61). Clock proteins are crucial for the proper functioning of the cell, especially in the case of cell growth/death, homeostasis, metabolism, and hormone release (60). When protein expression is disturbed, the CRs are also disrupted, which can amount to several disease states such as PCa (61). The mechanistic underpinnings of these proteins are still being studied and could provide profound insight into designing molecular therapies to treat cancers (62, 63).

**Table 1:** Genetic Involvement of the circadian system in GU cancers and clinical management options.

GU Cancers (Tumor Type)	Mechanisms of Disruption of Circadian Rhythms	Effects of Melatonin	Therapeutic Targets with Chronotherapy
*Prostate Cancer*	Downregulated PER2 and CLOCK (60)↓ Upregulated BMAL1 and CRY1 (60, 61),↑	Downregulates MMP-13 (109)	PBT (123) Docetaxel (115)
*Kidney Cancer*	Downregulated CLOCK, CRY1, CRY2, and BMAL1 (80)↓	Suppresses the Akt/MAPKs pathway (113) Downregulates MMP-9 (113)	Interferon-alpha (122) IL-2 (122)
*Bladder Cancer*	Downregulated BMAL1↓ Upregulated CLOCK and CRY1 (89)↑	Prevents the nuclear translocation of NF-κB (110) Induces apoptosis (110, 112)	Doxorubicin-cisplatin (122)

The role of the four clock proteins, BMAL1, CLOCK, PER, and CRY, were evaluated in relation to three GU cancers. The genetic effects of melatonin supplementation were explored as well as the primary therapeutic targets of chronotherapy to manage GU cancers.

The tumor microenvironment is a critical biological dynamic entity that merits exploitation in functional exchange with the external environment (light, temperature, specifically impacted by the circadian clock). EMT in solid tumors (including PCa) has been defined to play a significant role in cancer and a major contributor to metastasis (64). EMT is characterized by the loss of cell-cell adhesion, increased cell motility, and reduced E-cadherin expression, a structural adhesion molecule (65). E-cadherin, a calcium-dependent protein involved in cell-cell adhesion, is crucial for preventing PCa cells from migrating to bones to facilitate metastatic disease (66). Some several molecular mechanisms and pathways influence EMT, such as epidermal growth factor (EGF) and mitogen-activated protein kinase (MAPK) (67). Changes in signaling pathways ultimately alter the expression of transcription factors such as Snail and Zeb-1 (67). As a result of activation of these transcriptional repressors, E-cadherin expression levels are repressed, ultimately leading to enhanced mesenchymal and migratory markers in mesenchymal cells (68). Thus, EMT is functionally linked to promoting PCa metastatic progression, leading to stemness, therapeutic resistance, and ultimately lethal disease (68). Work from our group demonstrated that interconversion of EMT to mesenchymal-to-epithelial transition (MET) is observed in advanced PCa pre-clinical models in response to treatment with the second line taxane chemotherapy, cabazitaxel (52). This dynamically transient EMT-MET cycling allows cabazitaxel to prime the cells to retain a non-migratory phenotype, reducing the chances of metastasis (52). There is an ongoing effort to identify a temporal therapeutic window that can enable cells to overcome resistance by anti-androgen action (52).

Similar to phenotypic EMT navigating PCa, chronic CRs has been demonstrated to lead to the metastatic spread of breast cancer (65). CRs have a role in hormone expression and promote an immunosuppressive phenotype in endocrine-related cancers (69). Circadian-regulated transcription factors, such as PER2 and BMAL1, can regulate EMT through influencing EMT signaling effectors responsible for stemness and cell migration (69). Downregulated PER2 was associated with a higher likelihood of EMT in breast tissue, while downregulated BMAL1 decreased the invasion of mesenchymal cells in colorectal cancer (69). Melatonin was also found to regulate EMT and molecular pathways underlying the phenotypic conversion and cell invasiveness (65). MLT can activate GSK3β, an enzyme involved in cell proliferation, which reduces β-catenin levels, and subsequently leads to restoration of E-cadherin in human breast cancer cells (**Figure 1**) (65).

### Kidney Cancer

Kidney cancer accounted for nearly 431,300 cases worldwide in 2020 and has been increasing in recent years (70, 71). The median age of diagnosis is 65 years (72) (**Table 1**). Many tumors comprise kidney cancer, with 90% being RCC cases (73). Within the various molecular subtypes of RCC, clear cell RCC leads to the most deaths (73). The mortality rate of 30-40% for RCC is significantly greater than prostate and bladder cancers (74). Kidney cancer tends to be resistant to chemotherapy and radiation therapy, making immunotherapy the best option (75). With increased attention on potential mechanisms of progression such as angiogenesis and altered hypoxia signals, CRs research could explore ways to reduce the disease burden (76). Circadian pathways help maintain physiological fluctuations, such as water transport and essential renal function (77). Almost 43% of all protein-coding genes throughout the body showed CRs in transcription, many of them being in the kidney (77, 78). These gene expressions peak right before dawn and dusk (78). In a study linking the dysregulation of the circadian clock and RCC, clock genes were transcriptionally different in diseased versus healthy tissue (79). For example, CLOCK, CRY1, and CRY2 levels were downregulated in kidney cancer tissue (80). Patients that retained high levels of CLOCK had a better prognosis than those without (80). Like PCa, the clock proteins significantly predict the risk and progression of kidney cancer through intricate molecular mechanisms.

The clock proteins are crucial for regulating CRs and immune system function (81). The immune checkpoint pathway is suppressed when the clock protein BMAL1 is downregulated, causing sepsis (81). Sepsis and cancer share many immunological properties, so immunomodulatory agents could successfully treat both diseases (81). Increased expression of PD-1 and its ligand, PD-L1, help stimulate tumor-directed cytotoxic T cell function in both sepsis and cancer (81). The loss of the clock gene, BMAL1, showed increased PD-L1 expression in macrophages, which is associated with poorer sepsis survival (81).

### Bladder Cancer

Bladder cancer is ranked in the top ten most common cancers worldwide (82). Around 2.1% of cancer deaths are caused by urinary bladder cancer (UBC) each year, resulting in a high mortality rate (47). In Europe, the five-year survival rate for UBC was 68% (83). Unlike PCa, UBC has poorer outcomes within five years of being diagnosed. However, it has a higher survival rate than kidney cancer in Europe, which is 60% (83). UBC follows a similar prevalence trend of other GU cancer. It is less common in sub-Saharan Africa, India, and Mongolia and more common in Western Europe and Australia (84). The geographic distribution may be partly explained by exposure to tobacco, environmental pollutants, and occupational carcinogens, which are invariably linked to UBC incidence (85).

UBC can develop into either muscle-invasive bladder cancer (MIBC) or non-muscle-invasive bladder cancer (NMIBC) (86). For NMIBC, the course-of-treatment usually involves maintenance immunotherapy, whereas MIBC often requires chemotherapy (86). Combination chemotherapy provides good outcomes initially in impairing tumor growth, but it ultimately fails as cancer cells develop therapeutic resistance (87). Cisplatin is a first-line chemotherapy treatment that directly interacts with the circadian clock proteins and enhances the body’s natural response to cancerous cells (88). It upregulates CLOCK and BMAL1, resulting in increased proliferation and increased apoptosis, respectively (88). In bladder cancer tissue from human specimens, BMAL1 was downregulated, and CLOCK was upregulated, so cisplatin acts differently on both proteins through unclear mechanisms (89). Cisplatin has multiple opposing effects on tumor growth, resulting in stimulating pro-cancer effects (88). Thus our current understanding begs the question of interrogating the impact of disruption of circadian clock proteins on the molecular mechanisms underlying cell proliferation and apoptosis. In the context of contributing to therapeutic resistance, another clock protein, CRY1, was found to inhibit paclitaxel-induced senescence in bladder cancer cells (90). Typically, in urothelial tumors, CRY1 has been detected to be downregulated (89). While senescence causes cells to halt dividing, it also provides a way for cancer cells to become resistant to treatment (91). When the second-line therapy of paclitaxel is used, it prevents cell arrest and promotes the degradation of p53 (90). Healthy adults continually degrade p53, which is a tumor suppressor to stimulate p53 turnover (92). CRY1 is crucial in preventing the senescence induced by paclitaxel and delaying drug resistance (90).

## The Circadian Clock as the New Frontier to Overcome Therapeutic Resistance

### Melatonin Treatment

Melatonin (MLT) is a pineal gland hormone that can phase shift the SCN and provide timing information to the body (93). The pineal gland is crucial in regulating tumor growth and could become a target for therapeutics development (94). Melatonin levels naturally increase during dusk and taper off at dawn (95). Interestingly, subjects in perpetual darkness, such as visually impaired individuals, still display a 24.2-h cycle of melatonin and can have typical endogenous CRs (96).

The molecular mechanisms *via* which melatonin influences tumor cell proliferation and cancer metabolism are not clearly defined. Growing evidence suggests that melatonin may decrease the activity of endothelin-1 (ET-1), leading to downstream effects of downregulating hypoxia-inducible factor 1 alpha (HIF-1α) and VEGF, which both contribute to promoting angiogenesis (**Figure 1**) (97, 98). Preventing angiogenesis remains a critical goal to impair metastasis of kidney cancer (21). Significantly, it can also regulate breast cancer growth through two membrane melatonin receptors, MT1 and MT2, which are expressed in breast tissue, and impact survival signaling pathways (97). An overall decrease in melatonin levels has been associated with a higher risk of cancer, neurological disorders, and sleeping disorders (99). Thus, melatonin proves to be an effective and attractive therapy to improve the efficacy to toxicity ratio of anti-cancer drugs (100).

One of the most well-known hypotheses is that MLT is an epigenetic regulator that can prevent tumor growth by inhibiting telomerase activity and regulating linoleic acid uptake and metabolism, both crucial to proliferation (101). Circadian-dependent administration of MLT may confer tumor-suppressive properties (102). Melatonin has also been a potent, safe, and low-cost therapeutic in cancer research (103). A randomized controlled trial of solid tumors found that MLT reduces death by nearly a year (103). MLT also stimulates a robust chemotherapy response in palliative cancer care compared to receiving only supportive care (104). The patient’s quality of life is improved by reducing the side effects such as asthenia and thrombocytopenia (104). Thus, melatonin may enhance the therapeutic efficacy of patients with resistant GU cancers.

Despite the uncertainty that surrounds melatonin’s impact on cancer as a clinical disease, its protective benefits in human PCa are becoming increasingly evident. Men with high levels of urinary melatonin were less likely to develop advanced PCa (105). Advanced PCa is characterized by metastasis which involves tumor migration and invasion and ultimately lethal disease (106). Approximately 80% of patients with advanced PCa develop bone metastasis, a process that is linked with the expression of matrix metalloproteases (MMP) (107). Matrix metalloproteases are proteolytic enzymes responsible for breaking down connective tissue and allowing tumors to invade other tissues (108). MLT downregulates MMP-13 expression, which may suppress the metastasis of PCa (**Figure 1**) (109). MMP-13 is another excellent target for future therapeutic studies of PCa. It is of major significance to understand the molecular mechanisms driving the anti-tumor and anti-invasion properties of this agent.

MLT inhibits bladder and kidney cancer growth and metastasis (109). MLT prevents the nuclear translocation of NF-κB and decreases the expression of pro-inflammatory intermediates (**Figure 1**) (110). Recent studies have shown that MLT treatment resulted in increased apoptosis through NF-κB regulation in human gastric (111) and bladder cancer cells (110, 112). Moreover, MLT suppresses the Akt/MAPKs pathway and downregulates MMP-9, crucial for RCC progression (113). Through binding to the active site of MMP-9, MLT can arrest associated inflammatory signals that contribute to tumor growth (**Figure 1**) (114). Given the rapidly growing evidence at the mechanistic level, one could propose that MLT confers considerable transcriptional and post-translational control that are still not well understood.

### Chronotherapy

Chronotherapy involves orchestrating the timing of treatment administration to match the body’s endogenous CRs (115). This method has shown unequivocal success in tumor outcome and improved management of the disease (116). In addition, circadian dosing is crucial in limiting the toxicity of anti-cancer drugs and maximizing their efficacy (115). A characteristic example of an optimized (time-dependent response) is the first-line taxane chemotherapy, docetaxel, which is shown to have the best clinical outcome if administered in PCa patients between 6 and 9 am (115).

One must also consider that many cancer patients in late stages report having increased CRD with irregular sleep schedules (117). In breast cancer specifically almost 72% of advanced cancer patients display moderate-to-severe sleep disturbances (118). Chronotherapy could reduce the side effects of chemotherapy while also promoting a strong therapeutic response. In a retrospective study, patients undergoing high-dose radiotherapy for PCa in the evening had more GI complications than those in the morning (119). The toxicity of the drug is also decreased when administering the treatment in alignment with circadian oscillations. Lower toxicity levels could significantly relieve patients who have PCa, especially since GU cancer patients are older on average (119). There should also be a shift to similarly evaluating circadian-based dosing in therapy-resistant cancer patients. A circadian-modified infusion schedule can also allow clinicians to administer higher drug doses to induce a powerful response without the lethal toxicity. For example, patients with RCC could receive higher doses of floxuridine on a circadian-modified infusion schedule than on a continuous infusion schedule (120). This provides unique opportunities for a rigorous and impactful treatment of GU cancers while in their non-resistant phases for a better outcome. Chronotherapeutic schedules can also increase long-term survival and overall quality of life while on chemotherapies, such as oxaliplatin for metastatic colorectal cancer (121). In patients with metastatic UBC, treatment with doxorubicin-cisplatin resulted in a 57% objective response rate when coupled with chronotherapy (122). Other therapeutic options such as interferon-alpha and IL-2 (Interleukin-2) are promising agents to slow metastatic RCC, but they come at the risk of significant toxicity (122). By optimizing drug administration when toxicity would be minimized, clinicians can better use readily available compounds to treat GU cancers (122). Chronotherapy is not limited to only chemotherapy and immunotherapy in enhancing their treatment response outcomes. It can also be applied to radiation techniques, such as proton beam therapy (PBT), which directs smaller radiation doses at localized PCa (123). PBT was observed to have less severe lower urinary tract symptoms when given in the morning than in the afternoon (123).

Personalized medicine approaches can pave treatment strategies towards increasing patient survival and improving the quality of life for cancer patients. One may also consider that specializing current treatment methods according to a person’s chronotype, defined as a person’s preference for timing of sleep and activity, may lead to improved clinical outcomes. While chronotherapy has provided encouraging results in rendering cancer therapies more tolerable, more clinical studies are warranted. A significant issue is that much of the current research on chronotherapy in anti-cancer drugs do not have a strict time interval. Without a specific period, it is difficult for clinicians to administer treatment at the optimal time for maximum efficacy. Thus, there is an unmet need to functionally define the role of the CRs in cancer research.

### Environmental and Behavioral Interactions

Prior work in chronic disease patient populations suggests significant effects of environmental and behavioral interventions on reducing CRDs, including light therapies, physical activities, and diet modification which could, in turn, improve cancer patient outcomes (124, 125). Light is the strongest synchronizer of CRs, and exposure to ambient light at the right time could reduce CRDs and, thus, improve cancer patient physical and functional outcomes (126–130). Endocrine disruption due to exposure light during the circadian night has been implicated as carcinogenic, both in animal studies and in epidemiological studies in humans (131).

Evidence also suggests that physical activity could affect CRs (132–134). It has been shown that 1–3 hours of intense exercise can induce significant circadian phase shifts depending on the duration, intensity, and frequency of physical activities (132–134). Studies showed that early morning physical activities are associated with phase delays in the circadian clock (134, 135). However, early morning exercise offered protective effects for breast and PCa patients with an evening chronotype (136). Other studies showed that physical activities later at night induced phase delays in melatonin secretion (137). Individuals placed on prolonged periods of bed rest without exercise also show a circadian phase delay (125). Circadian misalignment is also observed when individuals participate in restrictive movement of one limb but not the other (125). This selective exercise leads to changes in the regulation of the clock genes, which are implicated in cancer pathways (**Figure 1**) (125). Additional assessment of the optimal time to exercise that can mitigate increased cancer risk and CRDs (124). One must note here that, while some studies show that exercise can alter circadian phase, its impact on the circadian clock is significantly less than the impact of light-dark patterns reaching the retina.

Lifestyle patterns in feeding/meal consumption (e.g., late-night meals) and diet programs (e.g., high fat diet) have been found to also influence circadian patterns in humans, although behavioral and sociocultural factors often control this (124). These circadian eating patterns are mirrored by both the gastrointestinal system, leading to rhythms in digestive secretions, gut motility, absorption of digested food, and blood nutrient concentrations (124). Feedback loops exist between the hormones controlling the circadian clock and those directing appetite and satiety, such as leptin, orexin, and ghrelin (124). Considering the roles of clock-related hormones, a food-entrainable circadian clock in humans may be present (124, 138, 139). Food-based entrainment enhances the synchronization of the peripheral and master clock, which can positively impact cancer regulation (124). Thus, in addition to understanding the impact of light exposure patterns, a further investigation into the interactive impact of exercise, diet, and nutrition on the risk, development, and clinical outcomes of GU cancers is likely to be impactful.

## Conclusion

A systematic review and meta-analysis of the previous studies in breast cancer female patients revealed a positive relationship between indicators of CRDs (e.g., nightshift work) and breast cancer risk (58). Changes in hormone secretion, caused by CRDs, was proposed as a contributing factor to the observed increase in breast cancer risk (58). Although breast cancer occurs predominantly in women, the biology and epidemiology of breast cancer share some similar features of GU cancer specially PCa (57, 58). For example, tumor progression in both breast cancer and PCa is strongly affected by sex hormones, which are, to a larger extent, influenced by CRDs and reduced amplitude of nighttime hormone melatonin.

The role of the CRs extends past currently known molecular regulations in transcription and translation. Given the extensive part of the four clock proteins (CRY, PER, BMAL1, and CLOCK), the circadian clock may regulate many cancer mechanisms such as apoptosis and therapeutic resistance (140, 141). Advanced GU cancers have poor outcomes and high mortality rates, making the development of therapeutic targets a time-sensitive task (142). A pioneering research study of circulating tumor cells, which are biomarkers of metastasis, has shown to follow specific circadian rhythmicity in animal models of PCa (143). By targeting PCa treatment to coincide when circulating tumor cells are at their highest concentration in the bloodstream, clinicians may be able to produce robust patient responses to treatment (143). Chronotherapy and MLT supplementation have also both proven to increase the efficacy of various chemotherapies and immunotherapies (121, 144). These are underused and beneficial tools that can diminish disease burden and progression.

Moving forward, the focus is the pursuit of CRs as defense mechanisms the body can engage to optimize therapeutic responses in patients diagnosed and treated for GU cancers. Circadian-based treatments can modulate the pharmacological ability of anti-cancer drugs towards improving therapeutic outcomes and be potentially incorporated into clinical trials for treatment optimization and improved patient survival. One may argue that the simple method of syncing drug administration with the body’s endogenous circadian clock can maximize the efficacy of clinically approved treatment strategies in managing advanced GU cancers. Moreover, the circadian clock provides an informative new platform about the optimal timing and dosing of the drug, compared to traditional pharmacokinetics and pharmacodynamics. Given the impact of the circadian clock on cancer progression and treatment response, the promise of enabling a viable defense against the GU tumors emerges. Driven by advanced technology, ongoing efforts from different centers focus on defining the roles of the clock proteins and their downstream effects in progression and clinical management of GU cancers to advanced disease. Thus whole-genome approaches, genomics, and proteomics would enable the detection of protein expression patterns and temporal networks of the clock proteins. Moreover, clinical studies implementing chronotherapy and melatonin supplementation are currently lacking in large patient cohorts ranked by their circadian profiles. The circadian-rhythms-navigated therapies pave the way for more effective implementation of current treatment modalities, their optimization towards overcoming therapeutic resistance and improving the quality of life in patients with GU malignancies.

## Author Contributions

Conceptualization: NM, PK, NK, MF. Resources: NM, MF, NK. Writing: PK, NM, MA, MF, MK. Figure preparation: MA, PK. Review and Editing: MF, NK. All authors contributed to the article and approved the final submitted version.

## Funding

This work was supported by the following funding: Grant # R01 CA232574/National Institutes of Health/NCI (NK); Grant #R01OH01668/NIH/NIOSH (MF); Department of Defense W81XWH-17-1-0590 #PC160194 and the National Institute of Nursing Research (1R21 NR0165)18-01A1 (NM).

## Conflict of Interest

The authors declare that the research was conducted in the absence of any commercial or financial relationships that could be construed as a potential conflict of interest.

## Publisher’s Note

All claims expressed in this article are solely those of the authors and do not necessarily represent those of their affiliated organizations, or those of the publisher, the editors and the reviewers. Any product that may be evaluated in this article, or claim that may be made by its manufacturer, is not guaranteed or endorsed by the publisher.

## Abbreviations

ADT, androgen deprivation therapy; Akt, protein kinase B; MAPKs –mitogen-activated protein kinase; AR, androgen receptor; BMAL1, Brain and Muscle ARNT-Like; CCGs, clock-controlled genes; CRDs, circadian rhythms disruption; CR, circadian rhythms; CLOCK, Circadian Locomotor Output Cycles Kaput; CRPC, castration-resistant prostate cancer; CRY, cryptochrome; EGF, epidermal growth factor; EMT, epithelial-to-mesenchymal transition; ET-1, endothelial-1; GSK-3β, glycogen synthase kinase-3β; GU, genitourinary; HIF-1α, hypoxia-inducible factor 1 alpha; IL-2, interleukin-2; MAPK, mitogen-activated protein kinase; MET, mesenchymal-to-epithelial transition; MIBC, muscle-invasive bladder cancer; MLT, melatonin; MMP, matrix metalloprotease; NMIBC, non-muscle-invasive bladder cancer; PBT, proton beam therapy; PCa, prostate cancer; PER, period; RCC, renal cell carcinoma; REV-ERBα (NR1D1), nuclear receptor subfamily 1 group D member 1; RORα, retinoid-related orphan receptor alpha; RORE, retinoid-related orphan receptors response elements; SCN, suprachiasmatic nuclei; TTFL, transcriptional and translational feedback loop; UBC, urinary bladder cancer; VEGF, vascular endothelial growth factor.
